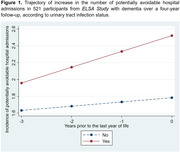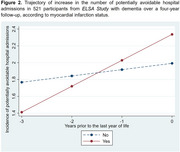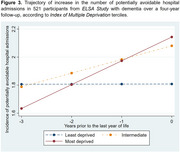# Which factors best discriminate increases in potentially avoidable hospital admissions as people living with dementia approach the end of life? Evidence from the ELSA Study linked to Hospital Episode Statistics

**DOI:** 10.1002/alz70861_107911

**Published:** 2025-12-23

**Authors:** Thaís Barros Pereira da Silva, Natalia Cochar‐Soares, Mariane Marques Luiz, Thales Batista de Souza, Sara Souza Lima, Jassiely Priscila de Faria Santos, Roberta de Oliveira Máximo, Feifei Bu, Andrew Steptoe, Cesar de Oliveira, Tiago da Silva Alexandre

**Affiliations:** ^1^ Federal University of São Carlos, São Carlos, São Paulo Brazil; ^2^ University College London, London, Greater London UK

## Abstract

**Background:**

Individuals living with dementia often experience increasing rates of potentially avoidable hospital admissions as they approach the end of life, following complex and poorly understood trajectories. There remains a critical gap in understanding the time‐dependent factors that discriminate individuals at higher risk of unplanned hospital use. We aimed to identify which factors are associated with increases in the number of potentially avoidable admissions in people with dementia in the final years of life.

**Method:**

Retrospective longitudinal study with a four‐year follow‐up, including 521 individuals aged ≥ 60 years from the *English Longitudinal Study of Ageing* (*ELSA Study)* with a clinical diagnosis of dementia, who had at least one potentially avoidable admission at the baseline. Participants’ data were linked to *Hospital Episode Statistics (Admitted Patient Care)* and death records from the *Office for National Statistics*. The outcome was the annual count of potentially avoidable hospital admissions, categorized as emergency admissions by hospital providers, defined as those occurring unpredictably and at short notice due to clinical need. This study included four annual time points, with the baseline defined as the last year of life (Year 0) and retrospective follow‐up across the preceding years (Years ‐1, ‐2, and ‐3). Generalised linear mixed models were performed to analyse the increase in the number of potentially avoidable hospital admissions, considering socioeconomic and clinical characteristics as exposure variables.

**Result:**

The increase in the number of potentially avoidable admissions in individuals with dementia was associated with urinary tract infections (0.137 admissions/year; 95% CI: 0.003–0.271), myocardial infarction (0.233 admissions/year; 95% CI: 0.005–0.462), and residing in areas classified within the most deprived tercile of the Index of Multiple Deprivation (IMD) (0.169 admissions/year; 95% CI: 0.021–0.318).

**Conclusion:**

The mechanism underlying potentially avoidable admissions among people living with dementia appears to involve two main pathways: clinical factors, through decompensations in the cardiovascular and genitourinary systems, and socioeconomic factors, which may reflect inequalities in access to preventive care. Identifying risk profiles for increased unplanned hospital use in the final years of life could support the development of targeted care models to reduce unnecessary admissions and improve end‐of‐life care.